# Reproductive and postsurgical outcomes of infertile women with deep infiltrating endometriosis

**DOI:** 10.1186/s12905-022-01666-5

**Published:** 2022-03-21

**Authors:** Ning Zhang, Shugen Sun, Yunxi Zheng, Xiaofang Yi, Junjun Qiu, Xiaodan Zhang, Ying Zhang, Keqin Hua

**Affiliations:** 1 Department of Gynecology, Obstetrics and Gynecology Hospital of Fudan University, 128 Shenyang Road, Shanghai, 200090 China; 2 Shanghai Key Laboratory of Female Reproductive Endocrine Related Disease, 413 Zhaozhou Road, Shanghai, China

**Keywords:** Deep infiltrating endometriosis, Surgery, Reproductive outcome, Life and sex quality

## Abstract

**Background:**

This study aimed to summarize and analyze clinical characteristics and reproductive outcomes in postoperative deep infiltrating endometriosis (DIE).

**Methods:**

This retrospective cohort study included 55 reproductive-aged patients who were diagnosed with DIE, wished to conceive and underwent resection surgery at the Obstetrics and Gynecology Hospital, Fudan University, from January 2009–June 2017. Those with any plausible infertility factor or abnormalities in the partner’s semen analysis were excluded. Patient characteristics, preoperative symptoms, infertility history, intraoperative findings and reproductive outcomes were followed up and recorded. Risk factors for reproductive outcomes were identified for women who became pregnant versus those who did not by univariate logistic regression. Additionally, pre- and postoperative endometriosis health profile questionnaire-30 (EHP-30), Knowles–Eccersley–Scott Symptom questionnaire (KESS), Cox Menstrual Symptom Scale (CMSS) and Female Sexual Function Index (FSFI) scores were used to evaluate the effect of DIE surgery on quality of life.

**Results:**

The average age was 30.22 ± 3.62 years, with no difference between the pregnancy and nonpregnancy groups. The average follow-up time was 26.57 ± 14.51 months. There were 34 pregnancies (61.82%): 24 (70.59%) conceived spontaneously and 10 (29.41%) by in vitro fertilization (IVF). Twenty-eight patients (82.35%) had term deliveries. The interval between operation and pregnancy was 10.33 ± 5.6 (1–26) months. Univariate analysis showed that a lower endometriosis fertility index (EFI) score (EFI < 8) was a risk factor for infertility (OR: 3.17 (1.15–10.14), p = .044). For patients with incomplete surgery, postoperative gonadotropin-releasing hormone agonist (GnRHa) administration improved the pregnancy rate (p < 0.05). Regarding quality of life, there was significant improvement (p < 0.05) in the postoperative EHP-30, KESS and CMSS scores compared with preoperative scores in both groups. Although there was no obvious difference in FSFI scores, significant improvement in dyspareunia was observed (p < 0.05).

**Conclusions:**

Overall, the postoperative pregnancy rate of DIE patients was 61.82%. Surgical management of DIE for patients with complaints of pain and with pregnancy intentions was feasible and effective. Long-term expectant treatment should not be advised for patients with lower EFI scores (EFI < 8), and postoperative IVF–ET may be a good choice. More cases should be enrolled for further study, and randomized studies are required.

Endometriosis is a common gynecological pathology that can cause pelvic pain [1] and reproductive failure [2]. Furthermore, infertility is a common problem for patients with endometriosis, but the causative mechanisms are still not completely known. Current treatment options for endometriosis-associated infertility include surgery and assisted reproductive technology (ART) [2, 3]. Laparoscopic surgery is suggested to be beneficial for minimal to moderate diseases, and there is evidence to support the use of laparoscopic surgery to improve fertility [4].

Deep infiltrating endometriosis (DIE) is a type of endometriosis and is defined as an endometriotic lesion invasion depth of greater than 5 mm into the peritoneal surface. The most common locations of DIE are the rectovaginal septum, uterosacral ligaments, pararectal fossa, and rectum. There is little evidence of a clear connection between DIE and infertility, and the absolute benefits of surgery for DIE are unclear. Among women who wish to become pregnant, optimal management, such as surgery versus first-line ART, for patients with more severe endometriosis is strongly debated. For patients with DIE, the situation is more complicated because evidence is sparse and the risk of severe surgical complications must be considered. Recently, the European Society of Human Reproduction and Embryology (ESHRE) stated that there was no evidence to support the use of surgical management for DIE prior to ART to improve the pregnancy rate. However, it is accepted that women planning to become pregnant and suffering from pain may benefit from surgical management and have favorable outcomes in terms of pain [5].

Therefore, the primary aim of this study was to summarize and analyze the clinical characteristics and reproductive outcomes of women with postoperative DIE. The secondary aim was to analyze postoperative improvements in life and sex quality among DIE patients.

## Materials and methods

A total of 250 patients of reproductive age who were diagnosed with DIE and underwent resection surgery at the Obstetrics and Gynecology Hospital, Fudan University, from January 2009 to June 2017 were included. Fifty-five patients wished to conceive, and those with any plausible infertility factor or abnormalities in the partner’s semen analysis were excluded. This study was approved by the Ethics Committee of the Obstetrics and Gynecology Hospital of Fudan University. Oral informed consent was obtained from all patients.

Patients were retrospectively selected based on the following criteria: age ≤ 40 years, histologic confirmation of DIE, primary or secondary infertility, regular menstrual cycles (21–35 days), no menstrual bleeding abnormalities, no ultrasonographic or radiologic features suggestive of ovulation failure and no history of pelvic inflammatory disease. Data regarding the semen analysis of the woman’s partner were also collected, and abnormal results were considered an exclusion criterion.

Before surgery, all patients underwent a detailed history collection and an accurate physical and imaging examination to assess various parameters, such as the basal body temperature (BBT), and transvaginal ultrasound (TVUS) scans, magnetic resonance imaging (MRI) and enteroscopy or intravenous pyelogram (IVP) were performed when necessary. For all the patients, the entire surgical procedure was performed with minimally invasive surgical techniques by a skillful multidisciplinary team (MDT) of gynecologists and urological and colorectal surgeons. Endometriosis severity was ascertained intraoperatively using the revised American Fertility Society (rAFS) scoring system [6]. The endometriosis fertility index (EFI) was used to evaluate the patient’s fertility [7, 8].

Data on the clinical characteristics of the patients, including the patient’s age, surgical history, symptoms, pre- and postoperative medical treatment, surgical details, duration of intervention, and procedures performed, were collected and recorded. Reproductive outcomes, including the pregnancy rate, modality of conception (spontaneous/ART) and live birth rate, were investigated. Follow-up data were collected from hospital records and telephone interviews, and the patients who responded were asked to participate in a follow-up visit comprising a pelvic examination (including transvaginal scans) and TVUS. Additionally, detailed information regarding medical therapy after surgery, recurrence of symptoms, fertility, and pregnancy rate was obtained. An analysis of the obtained data was performed via comparison of women who became pregnant after surgery with those who did not.

In our study, recurrence of endometriosis was defined as newly developed dysmenorrhea or pelvic pain reaching the pretreatment level or worse or newly developed endometriomas with a minimum diameter of 2.0 cm on pelvic ultrasonography. Preoperative and postoperative scoring of the Knowles–Eccersley–Scott Symptom questionnaire (KESS), Cox menstrual symptom scale (CMSS), female sexual function index (FSFI) and endometriosis health profile questionnaire-30 (EHP-30) were used to assess the impact of surgery on digestive outcomes, dysmenorrhea, quality of sex and quality of life, respectively.

Statistical analysis was performed using Stata 14.0 Software (Stata Corporation). Percentiles, ranges, mean values and SDs were calculated for continuous variables, and percentages were calculated for qualitative variables. Variable distributions depending on the group were compared by univariate analysis (Fisher's exact test for qualitative parameters and the t test and Mann–Whitney test for continuous variables). The cumulative pregnancy rate was estimated using Kaplan–Meier curves, which were compared when appropriate using the log–rank test. Statistically significant differences were defined as those with p values < 0.05.

## Results

**Fig. 1:**
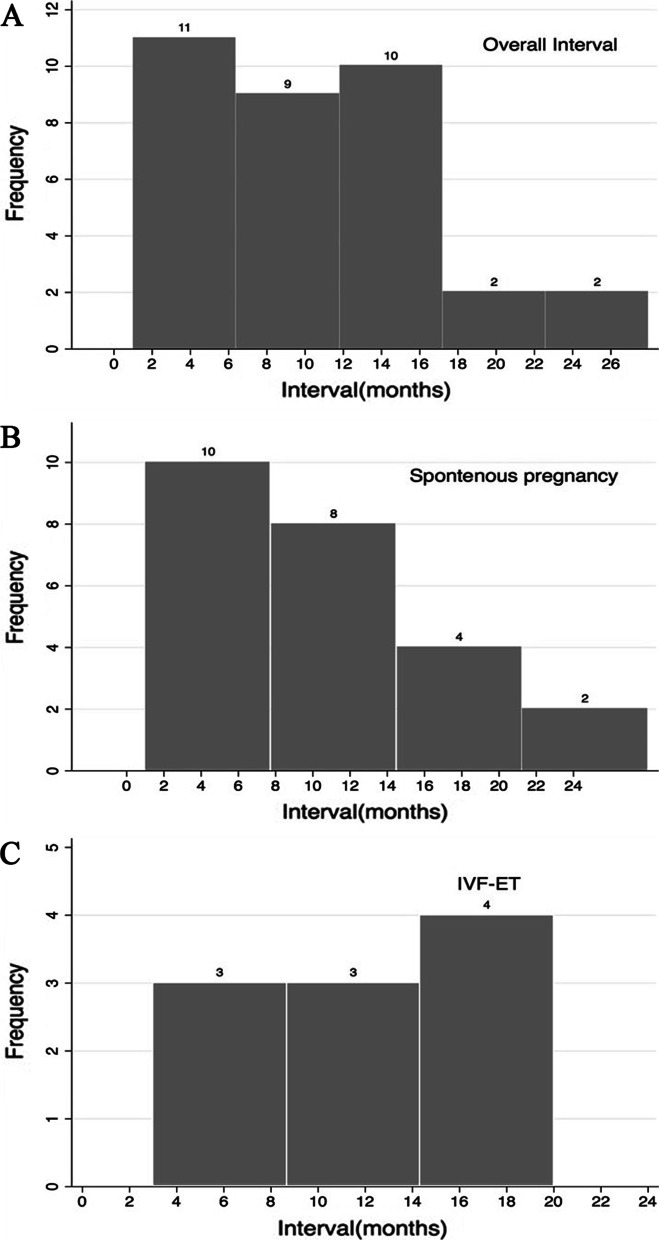
The interval between operation and pregnancy: **A** The overall interval between operation and pregnancy; **B** The interval for spontaneous pregnancy; **C** The interval for IVF–ET pregnancy

There were 34 pregnancies (61.82%): 24 (70.59%) that were spontaneous and 10 (29.41%) by in vitro fertilization (IVF). Twenty-eight patients (82.35%) had term deliveries, one had a missed abortion, 2 had spontaneous abortions, and 3 had induced abortions. The cesarean delivery rate was 35.71% with no preterm delivery, no neonatal admission to the intensive care unit and no fetal growth restriction (FGR) (Table 1). The average follow-up time was 26.57 ± 14.51 (12–71) months. The interval between operation and pregnancy was 10.33 ± 5.6 (1–26) months, with a mean conception time of 12.43 months when conceived by IVF and of 10.12 months when conceived spontaneously (Fig. 1).

**Table 1:** Reproductive outcomes and obstetric complications of 55 DIE patients with infertility

Parameter	Spontaneous pregnancy n = 24 (70.59%)	IVF–ET n = 10 (29.41%)	Total n = 34
Missed abortion	1 (4.17%)	0	1 (2.94%)
Ectopic pregnancy	0	0	0
Spontaneous abortion	1 (4.17%)	1 (10%)	2 (5.88%)
Induced abortion	3 (12.5%)	0	3 (8.82%)
Term delivery	19 (79.17%)	9 (90%)	28 (82.35%)
Cesarean delivery	6 (31.58%)	4 (44.44%)	10 (35.71%)
Preterm delivery < 37 w	0	0	0
Admission to NICU	0	0	0
FGR	0	0	0

DIE: deep infiltrating endometriosis; NICU: neonatal intensive care unit; FGR: fetal growth restriction

To determine the relative factors influencing postoperative pregnancy, we analyzed the obtained data by comparing women who became pregnant after surgery with those who did not. The basic information was comparable between the two groups (Table 2). The mean (± SD) ages were 30.2 ± 3.09 and 30.5 ± 3.65 years in the nonpregnancy and pregnancy groups (p = 0.76), respectively. A total of 47.6% and 69.7% of the patients had ovarian endometriosis in the nonpregnancy group and pregnancy group (p = 0.15), respectively, with no difference in endometrioma size (2.92 ± 2.95 vs. 3.69 ± 2.64, p = 0.36). A minority of patients had adenomyosis in both groups (14.3% vs. 26.5%, p = 0.28), and all patients with uterine adenomyosis underwent GnRHa treatment. A total of 9.4% and 20.6% of patients had previous endometrioma surgery, respectively (p = 0.72). Additionally, there was no significant difference in the preoperative CA125, FSFI, EHP-30, CMSS or KESS score between the groups (p > 0.05).

**Table 2:** Basic information of the 55 DIE patients with infertility

Parameter	Postoperative pregnancy		p value
	No	Yes	
Age (years)	30.2 ± 3.09	30.5 ± 3.65	.76
Ovarian endometriosis (n %)	10 (47.6%)	23 (69.7%)	.15
Size of endometrioma (cm)	2.92 ± 2.95	3.69 ± 2.64	.36
Adenomyosis (n %)	3 (14.3%)	9 (26.5%)	.28
Preoperative CA125 (U/ml)	81.98 ± 96.89	83.6 ± 81.1	.96
CA125 (> 35U/ml, n %)	18 (85.7%)	30 (88.2%)	.55
Previous endometrioma surgery	3 (9.4%)	7 (20.6%)	.72
Preoperative FSFI score	25.69 ± 3.1	26.32 ± 3.1	.48
Preoperative EHP-30 score	138.73 ± 66.22	140.7 ± 58.43	.91
Preoperative CMSS score	20.1 ± 23.56	20.1 ± 17.91	.99
Preoperative KESS score	5.1 ± 7.79	3.06 ± 5.40	.26

DIE: deep infiltrating endometriosis; FSFI: Female Sexual Function Index; EHP-30: Endometriosis Health Profile Questionnaire-30; CMSS: Cox Menstrual Symptom Cale; KESS: Knowles–Eccersley–Scott Symptom questionnaire

The surgical details and univariate analysis comparing patients who became pregnant to those who did not are presented in Table 3. The pregnancy group had higher EFI scores, and more patients underwent cautery procedures in the nonpregnancy group (p < 0.05); however, there was no difference in the rAFS phase between the two groups. In the univariate analysis, a lower EFI score (EFI < 8) was found to be a risk factor for infertility (OR: 3.17 (1.15–10.14), p = 0.044). Additionally, there was no difference in the number or size of the lesion, lesion location (rectum, rectovaginal septum or uterosacral ligament), residual lesion, operation type, operation time, blood loss or postoperative usage of GnRHa between the pregnancy and nonpregnancy groups. However, among patients who underwent incomplete surgery, more patients with postoperative GnRHa therapy became pregnant than those with no postoperative drug (100% vs. 0%, p < 0.05) (Table 4). Seventy-five percent of women with spontaneous pregnancies and 90% of women with IVF–ET pregnancies used postoperative GnRHa. For both the patients trying to conceive naturally and those undergoing IVF–ET, postoperative pregnancy was not related to the postoperative usage of GnRHa (p = 0.42 & p = 0.56).

**Table 3:** Intraoperative and follow-up findings of DIE patients with infertility

Parameter	Postoperative pregnancy		OR (95% CI)	p value
	No	Yes		
Number of lesions (N)	1.5 ± 1.04	1.6 ± 1.1	1.09 (0.62 ± 1.94)	.75
Size of lesions (cm)	2.32 ± 1.3	2.14 ± 1.42	0.91 (0.59 ± 1.39)	.66
Surgeon assisted (N %)	10 (47.6%)	13 (39.4%)	0.715 (0.23 ± 2.15)	.55
Operation time (min)	110.5 ± 48.5	127.5 ± 69.27	1.00 (0.99 ± 1.01)	.33
Blood loss (ml)	100.9 ± 53.94	148.5 ± 146.87	1.00 (0.99 ± 1.01)	.17
Residual lesion	3 (14.3%)	5 (14.7%)	1.03 (0.22 ± 4.86)	.97
Lesion location				
Rectovaginal septum (N %)	7 (35%)	17 (50%)		.27
Utero-sacral ligament (N %)	5 (25%)	9 (26.5%)		
Rectum (N %)	6 (30%)	4 (11.8%)		
Urinary system (N %)	0	3 (8.8%)		
Peritoneum (N %)	2 (10%)	1 (2.9%)		
Surgical procedures				
Shaving excision (N %)	12 (57.1%)	30 (88.2%)		.006
Lesion segment with anastomosis (N %)	1 (4.8%)	2 (5.9%)		
Cautery (N %)	7 (33.3%)	1 (2.9%)		
rAFS phase				
I + II	9 (42.9%)	11 (32.4%)	1.56 (0.51 ± 4.83)	.43
III + IV	12 (57.1%)	23 (67.6%)		
EFI (< 8)	15 (71.43%)	15 (44.11%)	3.16 (1.15–10.14)	.04
Postoperative GnRH agonist (N %)	15 (71.43%)	27 (79.41%)	1.54 (0.43 ± 5.44)	.50
Numbers of postoperative GnRH agonist (N)	3.25 ± 2.14	3.15 ± 2.48	1.04 (0.81 ± 1.33)	.75
Relapse	4 (19.05%)	5 (14.71%)	0.89 (0.16 ± 5.11)	.89

DIE: deep infiltrating endometriosis; rAFS: the revised American Fertility Society (rAFS); EFI: endometriosis fertility index

**Table 4:** Correlation between GnRH agonist and reproductive outcomes in the complete and incomplete excision groups

	Residual lesion					
	Yes		p value	No		p value
	Postoperative pregnancy			Postoperative pregnancy		
	Yes	No		Yes	No	
Postoperative GnRH agonist (N %)	5 (100%)	0	.02	22 (75.9%)	15 (83.3%)	.72
Number of postoperative GnRH agonist (N)	3.8 ± 1.3	0	.03	3.3 ± 2.3	3.7 ± 2.3	.54

**Fig. 2:**
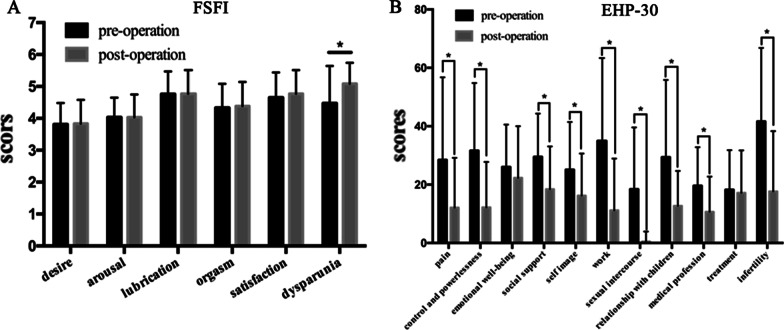
Comparison of pre- and postoperative FSFI and EHP-30 scores. *p < 0.05. FSFI: Female Sexual Function Index; EHP-30: Endometriosis Health Profile Questionnaire-30

The impacts of surgery on digestive outcomes, dysmenorrhea, quality of sex and quality of life are presented in Table 5. The results showed that there was significant postoperative improvement in the EHP-30, KESS and CMSS scores (p < 0.05). Regarding the EHP-30 scores, except for the emotional well-being and treatment aspects, all aspects were significantly improved postoperatively (p < 0.05). Although there was no obvious difference in the FSFI scores before and after surgery, a significant improvement in the postoperative scores of dyspareunia was observed compared with the preoperative scores (p < 0.05) (Fig. 2).

**Table 5:** Comparison of pre- and postoperative quality of life and sex of DIE patients

Parameter	Preoperation	Postoperation	p value
KESS	3.81 ± 4.9	2 ± 3.9	.04
CMSS	20.1 ± 19.97	8.67 ± 13.11	< .001
FSFI	26.1 ± 3.08	26.8 ± 3.19	.25
EHP-30	139.97 ± 60.81	81.1 ± 50.78	< .001

DIE: deep infiltrating endometriosis; FSFI: Female Sexual Function Index; EHP-30: Endometriosis Health Profile Questionnaire-30; CMSS: Cox Menstrual Symptom Cale; KESS: Knowles–Eccersley–Scott Symptom questionnaire

## Discussion

The situation is complicated and changeable for DIE patients with infertility. Additional factors, such as the patient’s age, ovarian function, and surgical history, need to be considered. DIE surgery can significantly reduce pain and improve quality of life [9, 10]. However, there is still controversy regarding whether surgery improves fertility. Our study evaluated the effect of DIE surgery on reproductive outcomes.

Most studies have reported that the overall pregnancy rate after DIE is 34–84.5% (CI 95 = 65.1–71.9%) [11–13]. In our study, the postoperative pregnancy rate among women with DIE was 61.82%, which is similar to most research results. A systematic review [14] showed that surgery for bowel DIE may improve the spontaneous pregnancy rate and have positive effects on IVF outcomes. However, surgical complications will extend the interval between surgery and pregnancy. Roman et al. [15] showed that the probabilities of achieving pregnancy after colorectal DIE surgery at 12, 24, 36 and 48 months postoperatively were 33.4% (95% CI: 20.6–51.3%), 60.6% (44.8–76.8%), 77% (61.5–89.6%) and 86.8% (72.8–95.8%), respectively. Surgery can also result in a high pregnancy rate for women with ureteral DIE [16, 17].

There are also several reports suggesting that DIE surgery can improve the pregnancy outcomes of ART. A retrospective cohort study showed that cumulative live birth rates were significantly higher for women who underwent first-line surgery followed by ART than for those who underwent first-line ART [18]. Soriano et al. [19] suggested that extensive laparoscopic surgery could improve IVF outcomes for patients with severe endometriosis and repeated in vitro fertilization failures. Similarly, Breteau et al. [20] discovered that infertile women with ≥ 2 IVF–ICSI failures should be referred for surgery and that the mean time from surgery to pregnancy is 11.1 months, which is similar to the median time to pregnancy of 12 months following attempts to conceive for all pregnancies. Therefore, surgery for DIE does not routinely delay conception.

DIE surgery is complicated, involves multiple organs and has a high risk of complications. Will the complications affect pregnancy outcomes? The answer to this question may influence the decision to perform DIE surgery for both doctors and patients. Ferrier et al. [21] analyzed the fertility outcomes of women who wished to conceive after a severe complication of surgery for colorectal endometriosis. The overall pregnancy rate was 41.2%, and 80% of women conceived spontaneously, which appeared satisfactory. The occurrence of a rectovaginal fistula, anastomotic leakage or deep pelvic abscess negatively impacts fertility outcomes. Therefore, patients with septic complications may benefit from rapid ART procedures. In our study, there were no serious complications. This may be because we have an experienced surgical team.

Considering the high risk of complications of DIE surgery, some researchers suggest first-line ART rather than surgery for DIE patients with infertility, especially those with tubal or male infertility factors [22]. However, the impact of DIE lesions on ART and obstetric complications is worth considering. A notable finding was that the number of DIE lesions was negatively correlated with ART outcomes [23]. Several studies have shown that DIE lesions can increase the risk of premature delivery, placenta previa, placental abruption and gestational hypertension. The cesarean section rate and the incidence of surgical complications (such as hysterectomy, peritoneal hemorrhage and bladder injury) in DIE patients were significantly higher than those in the normal group [24, 25].

There was no difference in rAFS scores between the pregnancy and nonpregnancy groups. The reason may be that the rAFS stages poorly reflect the severity of endometriosis-associated pain and infertility. Furthermore, the classification system has limited value in scoring DIE [26]. The ENZIAN classification has been recommended to classify DIE by the European Society of Gynecological Endoscopy (ESGE), ESHRE and the World Endometriosis Society [27, 28]. Additionally, the new #ENZIAN classification has been proposed and includes endometriosis of the peritoneum, endometriosis of the ovaries and the extent of adnexal adhesions, which makes up for the insufficiency of the ENZIAN classification [29]. However, the greatest challenge of the current classification systems seems to be their poor correlation with symptoms and infertility. Currently, we are completing the ENZIAN classification and analyzing its correlation with infertility, and the results will be presented in future articles.

The EFI has been proven to be a useful model for predicting pregnancy outcomes. Tomassetti et al. [30] discovered that the EFI is reliably reproducible and should be used for postoperative management and counseling of patients about their reproductive options. A systematic review and meta-analysis demonstrated that natural conception is the first choice for women with an EFI score of 6–10 [31]. In our study, we found that a lower EFI score (EFI < 8) was a risk factor for infertility. We suggest that patients with lower EFI scores (EFI < 8) should not be advised to undergo long-term expectant treatment and that postoperative IVF–ET may be a good choice. To explore this further, we performed a hierarchical analysis of EFI scores. We found that the least function (LH) scores of most patients (90.9%) were above four. Only 60% of DIE patients had ovarian endometriosis, and the anatomy and function of the fallopian tubes and ovaries were not seriously damaged. This may explain why the EFI scores were high for DIE patients with infertility. From another perspective, the mechanism by which DIE causes infertility is different from that of other endometriosis types.

AMH levels and antral follicular count (AFC) are considered valuable indicators of ovarian reserve. However, AFC has limitations in estimating the ovarian reserve of the ovary with the endometrioma [32]. The presence of a large endometrioma may impair the sonographic identification of small follicles adjacent to the cyst, and consequently, the ovarian reserve could be underestimated. Additionally, reports on the relationship between AMH and pregnancy in postoperative DIE are variable. Stochino-Loi et al. [33] found that preoperative AMH level did not significantly impact the probability of postoperative pregnancy when spontaneous conception and conception after ART were considered together. Reports of the relation between AMH or AFC and reproductive outcomes in postoperative DIE remain unconfirmed and is worth exploring in the future to help physicians and patients make clinical decisions.

In our study, there was no difference in residual lesions between the pregnancy and nonpregnancy groups. Qi Cao et al. [34] reported similar results. However, we discovered that there were more patients who underwent cautery surgery in the nonpregnancy group, which suggested that complete surgical excision of DIE is the first choice of surgical treatment. GnRH agonists are widely used in the treatment of endometriosis symptoms. However, it is controversial whether postoperative GnRHa improves DIE pregnancy outcomes. Although 79.41% of pregnant patients used postoperative GnRHa in our study, we discovered that the administration of postoperative GnRHa could improve the pregnancy rate only of patients with incomplete excision and not that of patients with complete excision. Bergenheim et al. [35] also found that infertile women with endometrioma(s) treated with radical surgery and long-term GnRHa downregulation immediately prior to IVF had a modest LBR after the first cycle, possibly due to immoderate suppression of ovarian function. Considering that the long-term use of GnRH agonists is associated with hypoestrogenic side effects and a substantial reduction in bone mineral density [36], our results suggest that postoperative GnRHa should be administered for patients with incomplete excision but that it is not necessary to routinely use postoperative medical treatment for patients with a reproductive desire who undergo complete surgical excision of DIE lesions.

Most patients in our study had significant improvements in gastrointestinal symptoms, dysmenorrhea and quality of life after DIE surgery. Although there was no obvious difference in FSFI scores, significant improvement in dyspareunia was observed. All these results indicated that DIE surgery could effectively improve pain symptoms. D’Alterio et al. [37] demonstrated that many medical and surgical treatments could demonstrate benefits in pain control and quality-of-life improvement. Overall, the surgical approach for severe DIE may be more effective and decisive. Therefore, surgery is required for DIE patients with obvious pain symptoms or clinically relevant intestinal or ureteral stenosis. Surgery might also be considered in young women who have repeated IVF failures [20]. Recent studies have also considered whether new technologies could improve surgical treatments for endometriosis; several have already found that the use of diode laser, plasma or CO2 lasers could improve pain symptoms and quality of life in selected cases [38, 39]. For DIE patients in our hospital, we used ultrasonic and plasma energy or cold scissors in nodule excision procedures, which also had feasible and effective results. More new energy instruments are worth exploring. However, some researchers suggest that surgery cannot be recommended for asymptomatic infertile women whose main goal is to treat infertility, as evidence to support such an approach is still scant. The use of an MDT comprising a gynecologist, reproductive specialist, urologist, colorectal surgeon, radiologist and counselor/psychologist is considered good practice in the management of endometriosis. Decisions should be tailored according to individual needs after the patient is provided with information on the potential benefits, harm, and costs of each treatment alternative [40, 41].

The major limitation of our study is related to its retrospective and nonrandomized design. In the context of infertility, only a randomized trial comparing first surgery to first ART could resolve this challenging issue. The sample size was not large enough to draw conclusions on some subgroups, such as patients who underwent incomplete surgery and lesion segments with anastomosis surgery. The strength of our study was that the baseline data of our patients were strictly matched. Rigorous prospective data were recorded by a dedicated clinical researcher who managed the follow-up of patients, with a very low dropout rate.

## Conclusion

Our study shows that DIE surgery could not only improve pain symptoms and quality of life but also achieve a satisfactory likelihood of reproductive outcomes, especially for patients with intolerable symptoms. Complete surgical excision of DIE is the first-choice surgical treatment. Administration of postoperative GnRHa is suggested for patients with incomplete excision. Patients with lower EFI scores (EFI < 8) should not be advised to undergo long-term expectant treatment, and postoperative IVF–ET may be a good choice for them. An experienced MDT and surgical team are necessary to formulate a satisfactory approach based on the patient's symptoms, expectations, and desire for pregnancy. Further randomized trials comparing primary surgery with first-line ART for DIE patients with fertility are needed.

## Data Availability

The datasets used and/or analyzed during the current study are available from the corresponding author on reasonable request.

## Abbreviations

DIE: Deep infiltrating endometriosis
ART: Assisted reproductive technology
ESHRE: European Society of Human Reproduction and Embryology
BBT: Basal body temperature
MRI: Magnetic resonance imaging
IVP: Enteroscopy or intravenous pyelogram
MDT: Multidisciplinary team
rAFS: Revised American Fertility Society
EFI: Endometriosis fertility index
FSFI: Female Sexual Function Index
EHP-30: Endometriosis Health Profile Questionnaire-30
CMSS: Cox Menstrual Symptom Cale
KESS: Knowles–Eccersley–Scott Symptom questionnaire
